# Factors predicting depressive symptoms in parents of children with autism spectrum disorder in eastern China

**DOI:** 10.1186/s12889-024-17731-7

**Published:** 2024-01-18

**Authors:** Xu Chen, Jiao Tong, Weijing Zhang, Xin Wang, Shan Ma, Dongdong Shi, Dongmei Yan, Yan Liu

**Affiliations:** 1 Lianyungang Maternal and Child Health Hospital, 669 Qindongmen Street, Haizhou District, 222000 Lianyungang, Jiangsu China; 2 National Health Commission Contraceptives Adverse Reaction Surveillance Center, NO.277 Fenghuang west Street, Gulou District, 210036 Nanjing, Jiangsu China; 3 Jiangsu Health Development Research Center, Nanjing, Jiangsu China; 4 Jiangsu Provincial Medical Key Laboratory of Fertility Protection and Health Technology Assessment, Nanjing, Jiangsu China

**Keywords:** Depressive symptoms, Parents, Children, ASD, Predictors, China

## Abstract

**Background:**

Parents of children with autism spectrum disorder (ASD) are at a higher risk of depression than parents of typically developing children and those of children with other developmental disorders. Depression affects the well-being and quality of life of parents of children with ASD and has serious consequences for the long-term health outcomes of children with ASD. Therefore, this study explored the current status of depressive symptoms in parents of children with ASD in eastern China and further analyzed multiple aspects of the predictors of depressive symptoms.

**Methods:**

A multicenter cross-sectional survey was conducted among parents of children with ASD in the rehabilitation department of a large specialized hospital and 10 rehabilitation centers for children with special needs in Lianyungang, Jiangsu Province, Eastern China. A structured questionnaire that focused on child-related factors, parent-related factors, depressive symptoms, courtesy stigma, and social support was used to obtain data. Binary logistic regression was used to identify the independent predictors of depressive symptoms in parents of children with ASD.

**Results:**

A total of 409 parents of children with ASD were recruited, of whom 18.8% had depressive symptoms. Parents of children with ASD who raised a child who spoke few to no words (odds ratio [*OR*]: 2.747, 95% confidence interval [*CI*]: 1.026–7.357), claimed a high economic burden (*OR*: 3.215, 95% *CI*: 1.234–8.379), reported no change or increased severity of ASD in their children (*OR*: 2.518, 95% *CI*: 1.108–5.720), and those with a higher courtesy stigma score (*OR*: 1.189, 95% *CI*: 1.093–1.294) were more likely to have depressive symptoms. Conversely, parents of children with ASD who were employed (*OR*: 0.427, 95% *CI*: 0.201–0.907), satisfied with their current marital status (*OR*: 0.429, 95% *CI*: 0.221–0.834), and those with a higher social support score (*OR*: 0.973, 95% *CI*: 0.950–0.996) were less likely to have depressive symptoms.

**Conclusions:**

Depressive symptoms are common in parents of children with ASD in eastern China. Therefore, screening and intervention for depressive symptoms in parents of children with ASD is necessary, especially for those with high-risk factors.

## Background

Autism spectrum disorder (ASD) is a complex neurodevelopmental disorder characterized by limited, repetitive behavioral patterns or interests, limited social interaction, and communication impairment [1, 2]. In the United States, ASD is reported in approximately 1 in 59 children [3]. This has drawn increasing attention to children with ASD. It is worth noting that parents of children with ASD encounter a variety of challenges in caring for their children, such as behavioral problems, sleep problems, emotion regulation deficits, and cognitive impairment, which often lead to higher mental health risks, including an increased risk of depression [4, 5]. Parents of children with ASD generally have worse psychological bonding outcomes than parents of typically developing children and those of children with other developmental disabilities [6–8].

Studies have shown that 12.5–34.2% of parents of children with ASD have clinically significant depressive symptoms, and mothers of children with ASD are more than three times more likely to suffer from depression than normal adult population [9–11]. However, screening and treatment of depressive symptoms in parents of children with ASD is often neglected, and few parents are able to access or actively seek the required health care services [12]. Depressive symptoms in parents of children with ASD have potentially negative impacts on the individual and have serious consequences for the long-term health outcomes of children with ASD [5, 13, 14]. The role of parents of children with ASD as advocates for children, coordinators of services, and interveners plays a crucial role in the treatment of children [4, 15]. Interventions for children with ASD often need to be implemented consistently in school and home settings, requiring parents to change their own behavior and increase the time spent playing and communicating with their children [5]. However, this may be very difficult for a parent with depressive symptoms. Depressive symptoms can lead to a deterioration in parental impatience and emotional control, which can cause difficulties in managing the child’s behavior and an inability to apply the skills learned during the treatment process to daily child care, thus, affecting the effectiveness of parental intervention, participation, and implementation as well as child recovery [16–18]. Studies have demonstrated that parental depression and other symptoms can minimize children’s response to treatment and make them benefit less from treatment [19]. Addressing parental symptoms such as depression using psychotherapy or other helpful resources may result in a better response to treatment in children with ASD [20]. In addition, severe depressive symptoms in parents were risk factors for increased psychiatric problems in children with ASD during the COVID-19 pandemic [21]. Therefore, there is an urgent need to study the status and influencing factors of depressive symptoms in parents of children with ASD so as to develop targeted and effective intervention measures to reduce depressive symptoms. This may not only improve the mental health of parents of children with ASD and family well-being but may also have flow-through effects that ultimately improve children’s developmental outcomes [22].

In recent years, an increasing number of studies have focused on depressive symptoms in parents of children with ASD. Foreign studies have shown that children’s age, time of diagnosis, comorbidity, time interval since diagnosis, poor language function, sleep problems, and the severity of symptoms are significantly associated with depressive symptoms in parents of children with ASD [14, 23–26]. Parents’ education level, occupation, marital quality, knowledge, perceived stigma, family function, social support, self-efficacy, subjective burden, and challenging parenting experience significantly predict depressive symptoms in parents of children with ASD [5, 11, 22, 24, 27–31]. Chinese studies have shown that mothers of children with ASD in the low-functioning group have a significantly higher incidence of depressive symptoms than those of children in the high-functioning group [32]. The educational level of mothers of children with ASD is associated with depressive symptoms [33]. Most of the previous studies only analyzed a few aspects of the factors associated with depressive symptoms in parents of children with ASD, and the literature in China is very limited. Notably, analysis of the Chinese caregivers of children and adolescents with ASD and other developmental disorders showed that caregivers with higher family income had significantly lower levels of depressive symptoms [34]. Income was also a predictor of quality of life for mothers of children with ASD [35]. Recent studies have found that exercise has a positive effect on depression [36, 37]. However, few previous studies have explored the effects of these factors on depressive symptoms among parents of children with ASD. In addition, most studies have analyzed the effect of the severity of symptoms in children with ASD on parental depressive symptoms [14, 25]. However, how changes in the condition of the disease affect depressive symptoms is unclear. In the Chinese collectivist culture, where social identity and social acceptance are highly valued, many parents of children with ASD may be more sensitive to the social perceptions of their children with ASD [38]. Moreover, because Chinese culture focuses on the family roots of developmental disorders, many Chinese parents of children with ASD may be blamed for causing their children’s present condition [39]. These may make parents of children with ASD more prone to psychological problems. However, there are few studies on depressive symptoms in parents of children with ASD in the Chinese context. Insufficient awareness of depressive symptoms in the parents of children with ASD may hinder efforts to intervene early in children with ASD in China [32]. Therefore, we hypothesize that the above factors may be associated with depressive symptoms in Chinese parents of children with ASD.

We conducted a cross-sectional survey in Lianyungang, Jiangsu Province, Eastern China. We aimed to assess the current status of depressive symptoms in parents of children with ASD and to analyze the predictors of depressive symptoms from multiple aspects, such as child-related factors, parent-related factors, courtesy stigma, and social support. This will be beneficial for developing interventions for depressive symptoms in parents of children with ASD; furthermore, this will help children with ASD achieve the best treatment outcomes and improve the health of the entire family.

## Materials and methods

### Study design and participants

A multicenter cross-sectional survey was conducted from October 2022 to February 2023 in the rehabilitation department of a large specialized hospital and 10 rehabilitation centers for children with special needs in Lianyungang, Jiangsu Province, Eastern China. Fathers or mothers of children with ASD undergoing rehabilitation in these institutions were invited to participate in this study. Only one parent was invited for per child with ASD. The inclusion criteria for participants were as follows: (1) age greater than or equal to 18 years; (2) be the mother or father of a child aged less than or equal to 12 years with a definite diagnosis of ASD; (3) be able to understand the content of the questionnaire; and (4) be living with a child with ASD. The exclusion criteria for participants were as follows: (1) mental disorders with a definite diagnosis; and (2) children with ASD had other serious physical or neurological diseases. Parents of children with ASD who met the criteria and agreed to participate in this study were asked to sign an informed consent form and anonymously complete a hard copy of the questionnaire. Before participating in the study, parents of children with ASD were informed by the investigators about the purpose of the study, the process of the study, the confidentiality of their data, and their right to withdraw from the study at any time. Investigators distributed questionnaires on site and were responsible for guidance and interpretation.

### Sample size

The minimum sample size required for this study was calculated using the single-population proportion formula. Due to the lack of previous relevant studies at the study site, we used a prevalence of depressive symptoms of 50% (*p* = 50%), 95% confidence interval (*CI*), a margin error of 5%, and a non-response rate of 10% to obtain the largest possible sample size. The sample size required for the study based on the calculation was 423. Therefore, a total of 430 parents of children with ASD were recruited into this study; 21 parents who did not completely fill out the questionnaire were excluded. Finally, a total of 409 parents of children with ASD were included in this study, with a participation rate of 95.1%.

### Data collection

A structured questionnaire, developed through a literature review and expert consultation, was used to collect data. The questionnaire focused on child-related factors, parent-related factors, depressive symptoms, courtesy stigma, and social support. Child-related factors included the child’s sex, age, comorbidities (referring to children who currently have other medical conditions), duration of rehabilitation, and functional speech. Parent-related factors included age, sex, place of residence, occupation, educational status, family monthly income, satisfaction with marital status, challenges of caring for children with ASD, economic burden, changes in a child’s disease status, physical exercise, average time spent with the child per day, alcohol intake, and cigarette smoking. The term economic burden refers to the economic costs of rehabilitating children with ASD borne by the family. Changes in a child’s disease status refers to the changes in the disease status of children with ASD treated by rehabilitation.

Depressive symptoms were measured using the Patient Health Questionnaire-9 (PHQ-9) [40]. It is a commonly used depression screening tool that assesses the frequency of depressive symptoms in the past two weeks. The PHQ-9 consists of nine items, each scored on a 4-point Likert scale ranging from 0 (not at all) to 3 (almost every day). Total scores range from 0 to 27, with higher scores indicating more severe depressive symptoms. PHQ-9 total scores of 0–4, 5–9, 10–4, and 15–27 indicate no depression, mild depression, moderate depression, and severe depression, respectively [41]. The recommended cutoff for positive results on the scale is 10 points, and it has been validated in the primary care population (sensitivity = 0.74, specificity = 0.91) and among pregnant women in the community (sensitivity = 0.95, specificity = 0.89) [42–44]. Therefore, a cut-off value of 10 was used in this study. The PHQ-9 has been validated in healthcare settings in multiple countries, including among parents of children with ASD, and has good internal consistency, construct, and criterion-related validity [5, 33, 41–43, 45]. In the current study, it had a reliability coefficient (Cronbach’s α value) of 0.910, and the internal consistency would not have improved with the deletion of later scale items. Its validity was confirmed by exploratory and confirmatory factor analyses (comparative fit index [CFI] = 0.931, goodness-of-fit index [GFI] = 0.913, Tucker-Lewis index [TLI] = 0.905, and standardized root mean square residual [SRMR] = 0.045).

Courtesy stigma was assessed using the Perceived Courtesy Stigma Scale (PCSS), modified from the Devaluation of Consumer Families Scale (DCFS) [46]. The scale consists of seven items, each scored on a 4-point Likert scale ranging from 0 (strongly disagree) to 3 (strongly agree). Total scores range from 0 to 21, with higher scores reflecting greater stigma. The PCSS has been validated for parents of children with ASD and has good internal consistency [47, 48]. In the current study, it had a reliability coefficient (Cronbach’s α value) of 0.893. The confirmatory factor analyses for it were CFI = 0.952, GFI = 0.929, TLI = 0.923, and SRMR = 0.050.

Social support was measured using the Multidimensional Scale of Perceived Social Support (MSPSS) [49]. The scale consisted of 12 items, including three dimensions of family support, friend support and other support. Each item was scored using a 7-point Likert scale ranging from 1 (very strongly disagree) to 7 (very strongly agree). Total scores range from 12 to 84, with higher total scores indicating higher levels of perceived social support for individuals. This scale has been widely used in several countries to assess the adequacy of perceived social support by the parents of children with ASD [28, 32]. The reliability and validity of the Chinese version of the scale have also been confirmed in some studies [50, 51]. In the current study, it had a reliability coefficient (Cronbach’s α value) of 0.955. The confirmatory factor analyses for it were CFI = 0.952, GFI = 0.899, TLI = 0.932, and SRMR = 0.044.

### Data processing and analysis

The completed questionnaires were coded and entered into a database established using Epidata version 3.1 (EpiData Association, Odense, Denmark) software. The data were exported to SPSS version 21.0 (IBM Corporation, Armonk, State of New York) software for statistical analysis. Continuous data were described as means and standard deviations (SD), and categorical data as frequencies and percentages. Chi-square tests were used to assess differences in the proportions of categorical data. To assess differences in the means of continuous variables, *t*-tests were used. Variables that were statistically significant in univariate analyses were included in a binary logistic regression model to assess the independent effect of each variable after adjusting for potential confounders. Collinearity between independent variables was tested before performing binary logistic regression. The results showed that the variance inflation factor of each variable was less than 10, and the tolerance was much greater than 0.1. Therefore, collinearity between independent variables was not present. In the current study, all comparisons were two-sided, and all tests of statistical significance used a critical *p* value of 0.05.

## Results

### The status of depressive symptoms in parents of children with ASD

**Fig. 1:**
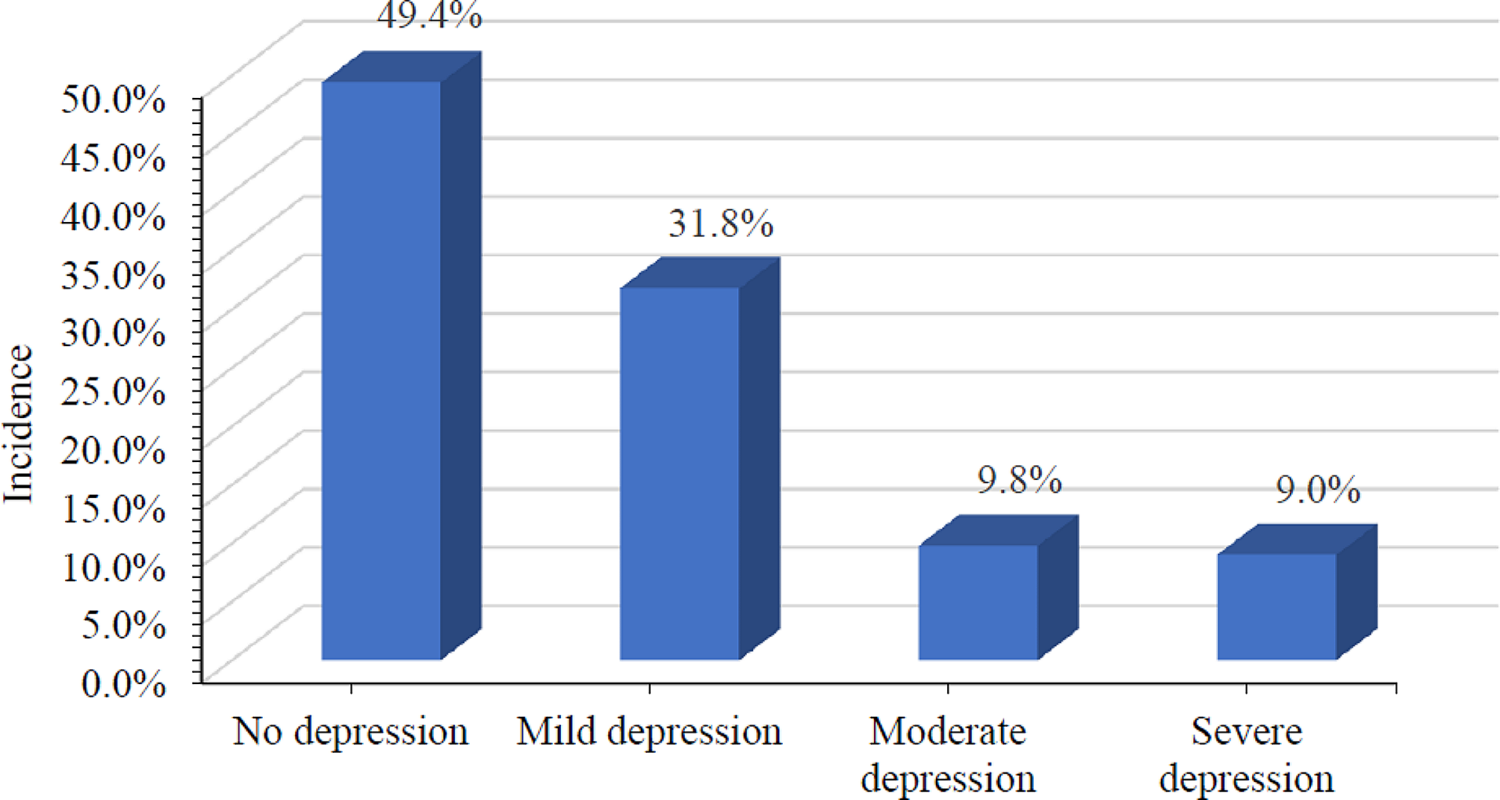
The status of depressive symptoms in parents of children with ASD

Based on their scores on the PHQ-9 scale, of the 409 parents of children with ASD, 130 (31.8%) had mild depressive symptoms, 40 (9.8%) had moderate depressive symptoms, and 37 (9.0%) had severe depressive symptoms. Using the recommended critical significant depressive symptoms value of 10, the incidence of depressive symptoms was 18.8% (Fig. 1).

### Child-related factors

The mean age of the children with ASD was 4.88 ± 2.30 years, and approximately two-thirds (66.7%) of the children were younger than 6 years. Most of the children (69.7%) were male, and a few (6.6%) had other medical conditions. More than half of the children (52.6%) had been in rehabilitation for more than one year, and nearly half (44.3%) spoke few to no words. Univariate analysis showed that the duration of rehabilitation and functional speech were significantly correlated with depressive symptoms (*p* < 0.05) (Table 1).

**Table 1:** Child-related factors and their association with depressive symptoms

Variable	Total n (%)	Depressive symptoms n (%)		χ^2^	*p*
		Yes	No		
Child’s sex				0.207	0.649
Male	285 (69.7)	52 (18.2)	233 (81.8)		
Female	124 (30.3)	25 (20.2)	99 (79.8)		
Child’s age				1.393	0.238
<6 years	273 (66.7)	47 (17.2)	226 (82.8)		
≥6 years	136 (33.3)	30 (22.1)	106 (77.9)		
Comorbidities				0.002	0.966
Yes	27 (6.6)	5 (18.5)	22 (81.5)		
No	382 (93.4)	72 (18.8)	310 (81.2)		
Duration of rehabilitation				5.819	**0.016**
≤1 year	194 (47.4)	27 (13.9)	167 (86.1)		
>1 year	215 (52.6)	50 (23.3)	165 (76.7)		
Functional speech				11.982	**0.003**
Few to no words	181 (44.3)	47 (26.0)	134 (74.0)		
Some words or phrases	136 (33.3)	21 (15.4)	115 (84.6)		
Sentences	92 (22.5)	9 (9.8)	83 (90.2)		

Significant values are in bold

### Parent-related factors

The mean age of the parents of the 409 children with ASD was 33.30 ± 5.10 years, and a large proportion of parents (65.3%) were 31–45 years old. Approximately two-thirds of the participants (63.8%) were mothers. More than half the participants (53.5%) lived in urban areas, and approximately half (49.9%) were currently employed. More than two-fifths of the participants (41.8%) had a college or higher degree, and only 17.8% of the participants had an average monthly family income of more than 10,000 yuan. Nearly a quarter of the participants (24.4%) were not too satisfied with their current marital status, and a large proportion (85.6%) believed that caring for children with ASD was a big challenge. Approximately one-third of the participants (34.2%) claimed that the economic burden of rehabilitation for their child with ASD was low, and only 13.9% of the participants reported that their child’s condition did not change or became more severe after treatment. More than half of the participants (55.0%) spent an average of 6 h or more with their children per day, and approximately one-fifth (20.3%) never exercised. More than one-fifth of the participants (22.2%) were current drinkers, and more than a quarter (26.9%) were current smokers. Univariate analysis found that place of residence, occupation, educational status, family income, satisfaction with marital status, challenges of caring for children, economic burden, changes in a child’s disease status, physical exercise, and average time spent with the child per day were significantly associated with depressive symptoms (*p* < 0.05) (Table 2).

**Table 2:** Parent-related factors and their association with depressive symptoms

Variable	Total n (%)	Depressive symptoms n (%)		χ^2^	*p*
		Yes	No		
Age				0.633	0.729
≤ 30	127 (31.1)	21 (16.5)	106 (83.5)		
31–45	267 (65.3)	53 (19.9)	214 (80.1)		
>45	15 (3.7)	3 (20.0)	12 (80.0)		
Sex				3.263	0.071
Mothers	261 (63.8)	56 (21.5)	205 (78.5)		
Fathers	148 (36.2)	21 (14.2)	127 (85.8)		
Place of residence				5.479	**0.019**
Urban	219 (53.5)	32 (14.6)	187 (85.4)		
Rural	190 (46.5)	45 (23.7)	145 (76.3)		
Occupation				15.189	**< 0.001**
Employed	204 (49.9)	23 (11.3)	181 (88.7)		
Unemployed	205 (50.1)	54 (26.3)	151 (73.7)		
Educational status				6.833	**0.009**
High school or below	238 (58.2)	55 (23.1)	183 (76.9)		
College or above	171 (41.8)	22 (12.9)	149 (87.1)		
Family income (RMB/month)				6.861	**0.032**
≤5,000	175 (42.8)	39 (22.3)	136 (77.7)		
5,001–10,000	161 (39.4)	32 (19.9)	129 (80.1)		
>10,000	73(17.8)	6 (8.2)	67 (91.8)		
Satisfaction with marital status				28.606	**< 0.001**
Satisfaction	309 (75.6)	40 (12.9)	269 (87.1)		
Not too satisfaction	100 (24.4)	37 (37.0)	63 (63.0)		
Challenges of caring for children				4.835	**0.028**
Big	350 (85.6)	72 (20.6)	278 (79.4)		
Small	59 (14.4)	5 (8.5)	54 (91.5)		
Economic burden				26.628	**< 0.001**
Low	140 (34.2)	7 (5.0)	133 (95.0)		
High	269 (65.8)	70 (26.0)	199 (74.0)		
Changes in a child’s disease status				14.066	**< 0.001**
No change or more severe	57 (13.9)	21 (36.8)	36 (63.2)		
Improvement	352 (86.1)	56 (15.9)	296 (84.1)		
Physical exercise				11.101	**0.004**
Often	35 (8.6)	4 (11.4)	31 (88.6)		
Sometimes	291 (71.1)	47 (16.2)	244 (83.8)		
Never	83 (20.3)	26 (31.3)	57 (68.7)		
Average time spent with the child per day				4.826	**0.028**
<6 h	184 (45.0)	26 (14.1)	158 (85.9)		
≥6 h	225 (55.0)	51 (22.7)	174 (77.3)		
Alcohol intake				0.070	0.792
Yes	91 (22.2)	18 (19.8)	73 (80.2)		
No	318 (77.8)	59 (18.6)	259 (81.4)		
Cigarette smoking				0.041	0.840
Yes	110 (26.9)	20 (18.2)	90 (81.8)		
No	299 (73.1)	57 (19.1)	242 (80.9)		

Significant values are in bold

### Courtesy stigma and social support

The average scores of courtesy stigma and social support were 7.48 ± 4.13 and 57.22 ± 13.55, respectively. Different courtesy stigma scores and social support scores significantly affected depressive symptoms in parents of children with ASD (*p* < 0.001) (Table 3).

**Table 3:** Parental courtesy stigma, social support and their association with depressive symptoms

Variable	Total (Mean ± SD)	Depressive symptoms (Mean ± SD)		t	*p*
		Yes	No		
Courtesy stigma	7.48 ± 4.13	10.17 ± 4.40	6.85 ± 3.82	5.746	**< 0.001**
Social support	57.22 ± 13.55	49.52 ± 13.03	59.00 ± 13.05	-6.104	**< 0.001**

Significant values are in bold

### Predictors of depressive symptoms

Binary logistic regression analysis revealed that parents of children with ASD who raised a child that spoke few to no words were almost 2.747 times more likely to have depressive symptoms than parents of children with ASD who could speak sentences (odds ratio [*OR*]: 2.747, 95% confidence interval [*CI*]: 1.026–7.357). Parents of children with ASD who reported a high economic burden were almost 3.215 times more likely to have depressive symptoms than parents of children with ASD who reported a low economic burden (*OR*: 3.215, 95% *CI*: 1.234–8.379). Parents of children with ASD who reported that their child’s disease status was unchanged or became more severe were almost 2.518 times more likely to have depressive symptoms than parents of children with ASD who reported that their child’s disease was improving (*OR*: 2.518, 95% *CI*: 1.108–5.720). Parents of children with ASD with higher courtesy stigma scores were more likely to have depressive symptoms (*OR*: 1.189, 95% *CI*: 1.093–1.294). However, employed parents of children with ASD were almost 0.427 times less likely to have depressive symptoms than unemployed parents of children with ASD (*OR*: 0.427, 95% *CI*: 0.201–0.907). Parents of children with ASD who were satisfied with their current marital status were almost 0.429 times less likely to have depressive symptoms than the parents of children with ASD who were not too satisfied with their current marital status (*OR*: 0.429, 95% *CI*: 0.221–0.834). Parents of children with ASD with higher social support scores were less likely to have depressive symptoms (*OR*: 0.973, 95% *CI*: 0.950–0.996). Thus, children’s functional speech, parents’ occupation, satisfaction with marital status, economic burden, perceived changes in a child’s disease status, courtesy stigma, and social support were predictive factors of depressive symptoms in parents of children with ASD (Table 4).

**Table 4:** Binary logistic regression analysis to determine the predictors of depressive symptoms

Variables	OR	95% CI	*p*
Duration of rehabilitation			
≤1 year	0.598	0.313–1.143	0.120
>1 year	1		
Functional speech			
Few to no words	2.747	1.026–7.357	**0.044**
Some words or phrases	1.778	0.657–4.815	0.257
Sentences	1		
Place of residence			
Urban	1.012	0.519–1.974	0.972
Rural	1		
Occupation			
Employed	0.427	0.201–0.907	**0.027**
Unemployed	1		
Educational status			
High school or below	1.363	0.648–2.868	0.415
College or above	1		
Family income (RMB/month)			
≤5,000	0.730	0.230–2.317	0.593
5,001–10,000	0.973	0.318–2.978	0.961
>10,000	1		
Satisfaction with marital status			
Satisfaction	0.429	0.221–0.834	**0.013**
Not too satisfaction	1		
Challenges of caring for children			
Big	1.247	0.413–3.767	0.695
Small	1		
Economic burden			
Low	1		
High	3.215	1.234–8.379	**0.017**
Changes in a child’s disease status			
No change or more severe	2.518	1.108–5.720	**0.027**
Improvement	1		
Physical exercise			
Often	0.508	0.133–1.933	0.320
Sometimes	0.615	0.306–1.236	0.172
Never	1		
Average time spent with the child per day			
<6 h	0.686	0.347–1.355	0.278
≥6 h	1		
Courtesy stigma	1.189	1.093–1.294	**< 0.001**
Social support	0.973	0.950–0.996	**0.021**

Significant values are in bold

## Discussion

Depression is a treatable mental health condition and should not be a barrier to parents optimally caring for their children with ASD [12]. This study assessed depressive symptoms in parents of children with ASD in eastern China and analyzed its predictors from multiple aspects. To the best of our knowledge, this study fills the gap in research related to depressive symptoms in parents of children with ASD in eastern China. The results of this study showed that the incidence of depressive symptoms in parents of children with ASD was 18.8%, which was lower than that in Hong Kong (25.4%) and Brazil (26.7%) and higher than that in the United States (12.5%) [9, 11, 14]. This may be due to differences in the study design and the sociocultural context. Traditional Chinese culture emphasizes shame and honor, which may cause people to pay too much attention to the perception and evaluation of others; this in turn leads to a higher incidence of depressive symptoms. Our findings suggest that depressive symptoms are common in parents of children with ASD in eastern China. Effective and targeted interventions to reduce depressive symptoms in parents of children with ASD are urgently needed. Therefore, identifying individual or environmental factors that may contribute to the alleviation of depressive symptoms in the parents of children with ASD is very important. This study found that children’s functional speech, parents’ occupation, satisfaction with marital status, economic burden, perceived changes in a child’s disease status, courtesy stigma, and social support were predictive factors of depressive symptoms in parents of children with ASD. Regarding child-related factors, this study showed that sex, age, comorbidities, and duration of rehabilitation were not significantly associated with depressive symptoms. Regarding parent-related factors, this study indicated that age, sex, place of residence, educational status, family income, challenges of caring for children, physical exercise, average time spent with the child per day, alcohol intake, and cigarette smoking were not significantly associated with depressive symptoms.

### Effect of child-related factors on depressive symptoms

The current study showed no significant association between the sex of children with ASD and parental depressive symptoms, which is consistent with the findings of previous studies [9, 23]. In addition, the current findings suggest that parents of children with ASD who raised a child who speaks few to no words are more likely to experience depressive symptoms. This is similar to the findings in previous studies that show an association between severe symptoms in children with ASD and a higher incidence of maternal or caregiver depressive symptoms, and a lack of functional language is particularly important in this regard [33, 52]. Mothers of children with ASD often have great expectations for their child’s language development and are likely to be depressed by the lack of language development [25]. The absence or lack of language function in children with ASD may also cause parents to be overwhelmed by their children’s behavior while caring for them, which may promote the occurrence of depressive symptoms. Previous studies paid more attention to the relationship between the severity of children’s symptoms and parental depressive symptoms [14, 25] and rarely analyzed the effect of functional speech on parental depressive symptoms. This study fills this lack of knowledge. Therefore, special attention should be paid to the parents of children with ASD who have no or little language function when developing interventions to reduce depressive symptoms in parents of children with ASD.

### Effect of parent-related factors on depressive symptoms

Caregiver occupation status was significantly associated with depressive symptoms [34]. Unemployment is an important predictor of depressive symptoms in parents or caregivers of children with ASD [29]. This study supports previous studies that found that parents of children with ASD who were employed were less likely to develop depressive symptoms. Because of caregiving responsibilities for children with ASD, parents are often forced to leave their jobs or to reduce their hours at work, which reduces family income and, thus, increases stress, leading to depressive symptoms [4, 53]. Therefore, much attention should also be paid to the parents of children with ASD who are unemployed. Relationship quality may be an important factor to be explicitly considered in an intervention paradigm for children with ASD [4]. Low marital satisfaction is associated with higher negative emotions in mothers of children with ASD [54]. This study also found that parents of children with ASD who were satisfied with their current marital status were more likely to be free from depressive symptoms. Marital satisfaction may buffer the effect of parental stress on depressive symptom; a good marital relationship may mitigate the effect of parental stress on depressive symptoms; and a poor relationship may exacerbate the effect of parental stress on depressive symptoms [4]. In addition, a positive marital relationship facilitates effective communication, facilitates problem solving, and increases the level of mutual support. Therefore, rehabilitation service providers for children with ASD should consider a family-centered approach to caring for children with ASD and how to improve parental relationships.

Parents of children with ASD have a substantial financial burden in terms of the costs of rehabilitation training and treating medical illnesses [55]. A survey conducted in China showed that children with ASD required higher costs to raise them than those with physical or mental disabilities [56]. In China, it was estimated that each family with a child with ASD spent at least RMB 30,000 per year on services for the child in rehabilitation institutions [33]. The current study showed that parents who perceived that the cost of rehabilitation for their children with ASD was a high economic burden on the family were more likely to have depressive symptoms. This is consistent with the findings in previous literature on the health-related effects of financial stress factors, which showed that financial hardship predicted anxiety in mothers of children with ASD [22]. Thus, financial support may play an important role in reducing depressive symptoms in the parents of children with ASD. At present, China has expanded its medical insurance coverage and optimized its reimbursement policy. It includes the treatment and rehabilitation of children with ASD in the medical insurance coverage and has increased the reimbursement proportion and limit. However, the rehabilitation and treatment of children with ASD requires long-term investment and support, and the joint efforts and support of all sectors of society are still required.

Previous studies have shown that children’s behavioral problems are reliable predictors of depressive symptoms in mothers of children with ASD [57]. Severe behavioral symptoms in children increase the likelihood of severe depressive symptoms in parents by 35 times [14]. The current study expands on previous findings that showed that parents who perceived no change or more severe illness in their children with ASD were more likely to be depressed than parents who perceived improvement in their children with ASD. Parents who perceived that their child’s disease was unchanged or became more severe were more likely to be confused about the future of the child and to have doubts about the care the child needs, which may in part increase the occurrence of depressive symptoms. Therefore, clinicians should increase communication with these parents to solve their confusion and doubts, thereby reducing the incidence of depressive symptoms. In addition, compared with some Western countries, there are relatively few ASD rehabilitation institutions and resources in China, which prevents some children with ASD from receiving timely and effective rehabilitation. This may affect the improvement of the disease, and more resources should be made available.

### Effect of courtesy stigma on depressive symptoms

Previous studies have found that vicarious and self-stigma are positively correlated with depressive symptoms in parents of children with ASD, and internalized stigma is significantly correlated with depressive symptoms in parents of children with ASD [11, 58]. The current study adds to previous research by clarifying that courtesy stigma is a risk factor for depressive symptoms in parents of children with ASD. The Chinese culture emphasizes group harmony. Some families of children with ASD may face social isolation due to their children’s abnormal behavior and communication style, which may cause significant stigmatization of these Chinese parents [11, 59]. In addition, the parents of children with ASD are also subject to unjustified criticism and accusations that they are passing on bad genes or providing ineffective parenting because of their biology and closeness [38]. Qualitative studies conducted in China have also shown that parents of children with ASD are criticized for failing to discipline their children or for poor parenting [60]. Therefore, there is an urgent need to develop effective anti-stigma interventions to reduce depressive symptoms in parents of children with ASD. Studies have shown that knowledge interventions and contact interventions can reduce the prejudice of community members and improve the public’s attitude towards children with ASD and their families, thereby reducing the stigmatization of parents of children with ASD [38].

### Effect of social support on depressive symptoms

Support and education should be provided to parents of children with ASD on an ongoing basis throughout their child’s development [9]. Strengthening social support can reduce depressive symptoms in mothers of children with ASD. Support from family members is an important component of social support, and interventions to improve family functioning may help address depressive symptoms in mothers of children with ASD [5]. The current study revealed that social support is a protective factor against depressive symptoms in parents of children with ASD. This is consistent with previous studies in which social support was shown to be a significant predictor of depressive symptoms in both mothers and fathers of children with ASD [31]. Social support is key to improving parental adaptability in the management of children with ASD [61]. Therefore, increasing social support is an aspect of concern in the development of depressive symptom interventions for parents of children with ASD.

### Limitations and suggestions for future research

Several limitations of the current study must be acknowledged. First, a causal relationship between the variables and outcome could not be established because this is a cross-sectional study. Future longitudinal studies are required to further evaluate the associations found. Second, fathers and mothers were recruited separately, and there were no matched parenting pairs. Future recruitment of both fathers and mothers of the same child with ASD is needed for better comparative analyses. Third, the current study was conducted in only one city, and due to the influence of socio-economic and cultural background, the results should be cautiously extrapolated to regions with different conditions. Future studies in different cultural contexts are needed. Finally, the measures of variables such as depressive symptoms in the current study were all based on self-report, with the possibility of bias. Multiple methods of data collection need to be considered in future studies.

## Conclusion

The results of this study showed that the prevalence of depressive symptoms in parents of children with ASD in eastern China was high, and children’s functional speech, parents’ occupation, satisfaction with marital status, economic burden, perceived changes in a child’s disease status, courtesy stigma, and social support were predictors of depressive symptoms in parents of children with ASD. Interventions that focus on depressive symptoms in parents of children with ASD need to be developed. In the formulation of intervention measures, efforts should be focused on reducing the risk factors and strengthening the protective factors of depressive symptoms to achieve optimal effectiveness of the intervention and the healthy development of children with ASD.

## Data Availability

The datasets generated and/or analysed during the current study are not publicly available for ethical reasons but are available from the corresponding author on reasonable request.

## Abbreviations

ASD: Autistic spectrum disorder
PHQ-9: Patient Health Questionnaire-9
CFI: Comparative fit index
GFI: Goodness-of-fit index
TLI: Tucker-Lewis index
SRMR: Standardized root mean square residual
PCSS: Perceived Courtesy Stigma Scale
MSPSS: Multidimensional Scale of Perceived Social Support
SD: Standard deviation
*OR*: Odds ratio
*CI*: Confidence interval
